# Effects of Rotary Swaging Parameters and Artificial Ageing on Mechanical Properties and Microstructure of 2024 Precipitation-Hardenable Aluminium Alloy

**DOI:** 10.3390/ma13010143

**Published:** 2019-12-30

**Authors:** Jan Nacházel, Jan Palán, Jaromír Dlouhý, Peter Sláma, Zbyšek Nový

**Affiliations:** COMTES FHT a.s. Průmyslová 995, 334 41 Dobřany, Czech Republic; jan.palan@outlook.com (J.P.); jaromir.dlouhy@comtesfht.cz (J.D.); peter.slama@comtesfht.cz (P.S.); zbysek.novy@comtesfht.cz (Z.N.)

**Keywords:** aluminium alloy, EN AW-2024, rotary swaging, artificial aging, EBSD, tem

## Abstract

This work concerns mechanical properties in relation to microstructural changes in hardenable EN AW-2024 aluminium alloy in wrought and heat treated condition. The treated material benefits from synergistic effects of hardening mechanisms. Grain boundary strengthening and work hardening were activated in this material by rotary swaging. Rotary swaging is a method which shows great promise for industrial use. Precipitation hardening was achieved thanks to the material’s age hardening ability. First, the material was artificially-aged in a furnace at 140–180 °C. Second, natural ageing was used. Mechanical properties of the as-treated material were tested and microstructural processes were explored using electron microscopy and differential scanning calorimetry. The treatment route which delivered the best results was as follows: solution annealing 500 °C/1 h + water cooling + rotary swaging + artificial ageing 160 °C/21 h. This led to a yield strength close to 640 MPa, and ultimate strength above 660 MPa, and elongation of 8%. Electron backscatter diffraction observation revealed that in this condition, the ratio of Low-angle to High-angle grain boundaries is 80:20. The microstructure contains both T-phase in the interior of grains, whose particles are normally oval-shaped, and S-phase, which is present in two shapes: small oval particles or coherent needles aligned to <100> direction.

## 1. Introduction

In EN AW-2024 aluminium alloy, the principal alloying elements are Cu and Mg. It is a wrought age-hardenable alloy. Hardening occurs through the process of ageing, whether natural or artificial. Ageing involves a sequence of transformations described by Bagaryatsky in the 1950s [1]:

SSSS–GPB zones–S″–S′–S

Here SSSS stands for super-saturated solid solution. GPB is an abbreviation of Guinier-Preston-Bagaryatsky zones, a structure analogous to GP zones in Al-Cu systems. Using the term GPB zones, Silcock [2] referred to atomic-scale zones proposed by Bagaryatsky. The latter believed that these zones were clusters of Cu and Mg atoms with a short-range order. In more recent studies, the term “Cu-Mg clusters” was adopted [3]. Nevertheless, no readily-apparent difference between these phases has been identified, such as the shape, composition, arrangement, and orientation of their structure [4]. S″ is a phase which is believed to differ from GPB zones as well as from the equilibrium phase S or S′. A number of possible structures of this phase were proposed [5,6]. Some authors, including [7,8], suggested and worked to prove the existence of what they referred to as GPB2 zones. In any case, the structure of this phase, which is believed to be fully coherent with the matrix, has not been established definitely [4]. One of the probable forms of this phase is Al_5_(CuMg)_3_ [9]. Phase S′ has the same structure as S but a different lattice parameter, and therefore a different degree of coherency. Today, distinguishing between these phases is not considered necessary. The sequence is therefore reported with only S phase [4]. S is an equilibrium phase incoherent with the Al matrix. Its composition is Al_2_CuMg. Numerous studies have explored the structure of this phase. The model which gained the widest acceptance is the Perlitz–Westgren model, which postulates that it has the orthorhombic structure with lattice parameters of a = 0.400 nm, b = 0.923 nm, and c = 0.714 nm [4]. Recent publications refer to the following sequence of transformations [9]:

SSSS–Cu-Mg clusters – GPB2–S

Essentially, as follows from the above overview, it is still the same sequence under a different name. Simply, the ageing sequence comprises three phases:

- Clusters (zones) of atoms with a short-range order.
- Non-equilibrium phases coherent with the matrix.
- Equilibrium phases incoherent with the matrix.

Precipitates cause precipitation hardening, which is one of strengthening mechanisms seen in materials which exhibit partial solubility in solid state, and therefore develop secondary phases. The size of peak-aged precipitates, i.e., when the precipitates are near the thermodynamic equilibrium [4], is sufficient to prevent their shearing, while, at the same time, the particles are dense enough to stop dislocation movement [10]. These phases are major obstacles to dislocation movement, and therefore contribute strongly to the final mechanical properties of the material.

The microstructure of metals can be profoundly changed by another method as well: forming. Forming processes involve work hardening and grain size strengthening. Methods which can produce ultrafine-grained (UFG) or nanocrystalline structures continue to be explored today. Forming processes belong to the Top-Down group of grain refining techniques, such as severe plastic deformation (SPD) processes including equal channel angular extrusion (ECAP) [11,12,13] and high pressure torsion (HPT) [14,15] or non-SPD process rotary swaging (RS) [16,17].

Although RS was developed more than 60 years ago. Yet, it is still rarely used for producing UFG materials, as evidenced by the scarcity of publications on this topic. RS introduces strain by repeated blows by swages. The advantages of this method consist primarily in the relatively high speed of processing, good quality of surface and its compatibility with industrial use. Its drawbacks include texture, which is produced in the material and leads to anisotropy. Despite that, it is a promising technique, as shown in [18,19].

EN AW-2024 alloy has been used as a material for airframes for decades, primarily thanks to higher fatigue resistance than in other aluminium alloys [20]. Drawn tubes and extruded bars from this material are converted into parts of airplane seats and structural parts. Products of this kind must possess good mechanical properties.

The aim of this study was to find a method of processing EN AW-2024 aluminium alloy leading to unique mechanical characteristics (combination of strength properties and ductility), and describe the microstructures in such material.

## 2. Materials and Methods

Experiments in this study were carried out on EN AW-2024 material in T351 condition. Its chemical composition is given in Table 1. They were extruded bars 12 mm in diameter.

**Figure 1:**
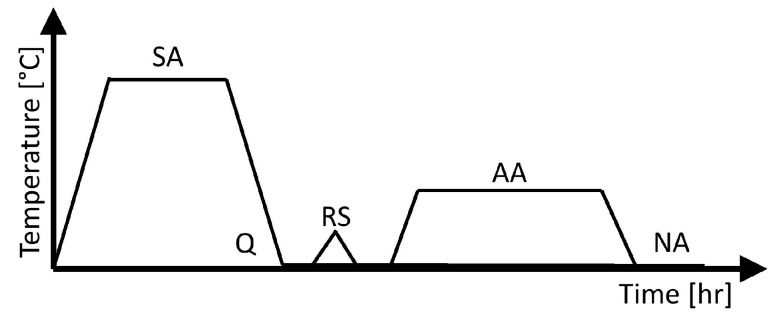
Schematic representation of the basic experimental treatment.

The material was processed using a sequence which is depicted schematically in Figure 1. It involved solution annealing (SA) followed by quenching (Q). The next step was RS. Since forging introduces appreciable deformation heat, RS was followed by cooling. The last step was artificial ageing (AA) or natural ageing (NA).

The SA temperature and time at temperature were 500 °C and 1 h. Q after SA was first carried out in water at room temperature (designated W). Second, Q in liquid nitrogen (designated N) was performed as well. RS followed immediately after Q. RS was applied in three sequences out of the total four. The first sequence involved no RS (designated as 0RS). The stock diameter prior to forging was 12 mm. In the second sequence (designated as ½RS), one forging pass reduced the diameter to 11.6 mm. In the third sequence (designated as 1RS), the diameter was reduced to 11.4 mm in one forging pass. In the fourth one (designated 2RS), two passes were used, reducing the diameter to 11.4 mm, and then to 10.3 mm. Individual parameters of the RS process are summarised in Table 2.

The AA temperatures were from 140 to 180 °C and the times at temperature varied from 1 to 29 h for the highest AA temperature and to 168 h for the lowest AA temperature. The experimental treatment led to several different conditions of the material whose designation codes followed the X-Y-Z pattern where:

- X identifies the cooling medium which was used after SA (W/N),
- Y indicates the amount of deformation introduced by RS (0RS/½RS/1RS/2RS),
- Z is the AA temperature or NA (180/160/150/140 °C/NA),

The instrument used for scanning electron microscopy (SEM) electron backscatter diffraction (EBSD) analysis was Jeol JSM-IT500HR (JEOL Ltd., Tokyo, Japan) with a Schottky cathode and an EBSD system from EDAX. EBSD maps were collected at an acceleration voltage of 30 kV. The image field size was 85 µm × 65 µm, the step size being 0.2 µm. The EBSD data was evaluated using IOM Analysis software (OIM Analysis™ v8, EDAX, Leicester, UK). The grain boundaries with a misorientation between 2° and 15° were considered low-angle grain boundaries (LAGB), whereas those with a misorientation higher than 15° were classified as high-angle grain boundaries (HAGB). Grain boundaries with a misorientation angle less than 2° were excluded from the evaluation in all material conditions due to orientation noise.

Transmission electron microscope Jeol 2100F (JEOL Ltd., Tokyo, Japan) was employed with an acceleration voltage of 200 kV. Micrographs were taken using the STEM mode. Energy-dispersive X-ray spectroscopy (EDS) maps were collected with X-Max80 detector from Oxford Instruments (Abingdon, UK) combined with Aztec software (Aztec Software, Springfiel, IL, USA). Alternatively, transmission electron microscopy (TEM) observation was performed with FEI Titan Themis 60-300 cubed, at an acceleration voltage of 300 kV. STEM mode was used with an High-angle annular dark field detector.

Foils were prepared using two different techniques in order to eliminate the effect of foil preparation on the microstructure. One of them was electrochemical etching using Tenupol 5 device (Struers, Willich, Germany). The etching reagent was a 30% solution of HNO_3_ in methanol. The etching temperature was −20 °C. The other foil preparation technique was the conventional argon ion polishing using a Gatan PIPS device (GATAN Inc., Pleasanton, CA, USA).

The tool used for differential scanning calorimetry (DSC) analysis was a DSC PT1600 calorimeter from Linseis (Selb, Germany). An S-type sensor and nitrogen protective atmosphere were used. The specimens weighed approx. 25 mg. They were placed in alumina crucibles.

Tensile specimens were tested in a 250 kN-capacity Zwick machine (ZwickRoell, Ulm, Germany). For each material condition, two specimens were produced and tested. They were round specimens with a gauge section 6 mm in diameter and 40 mm in length. M10 threads on their heads were used for clamping in the machine. The test velocity was constant: 2 mm/min. A mechanical extensometer with an initial length of 25 mm was used for strain measurement.

## 3. Results

### 3.1. Effects of Cooling Rate

**Figure 2:**
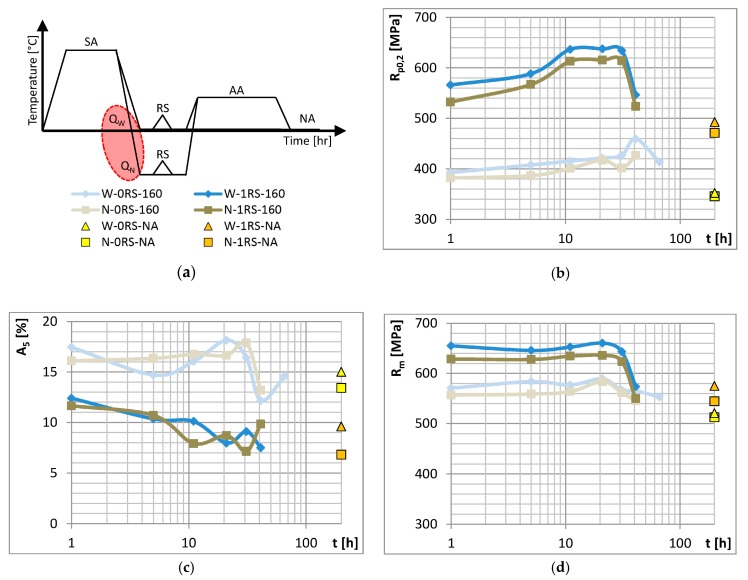
(**a**) Schematic representation of the cooling rate experiment and its results: (**b**) yield strength (**c**) elongation and (**d**) ultimate strength vs. time of AA (the time of NA was 1 month but the operation is indicated at t = 200 h).

Effects of cooling rate was studied on specimens after experimental treatment (as shown schematically at top left in Figure 2) which involved different media for quenching after SA. One half of all specimens were quenched in water (25 °C) and the other in liquid nitrogen (−196 °C).

Figure 2 shows ageing curves. All the curves, except Nitrogen-0RS-160, consist of three portions which indicate different stages of ageing: under-ageing, peak-ageing and over-ageing. The rate of cooling after SA and the temperature to which the specimen had been quenched had no significant effect of the mechanical properties in question. In other words, the rate of cooling achieved by water quenching is supercritical. Increasing this rate or decreasing the final temperature had no significant impact on the properties. Slightly better mechanical properties were found in water-quenched specimens.

### 3.2. Effects of Strain Magnitude

**Figure 3:**
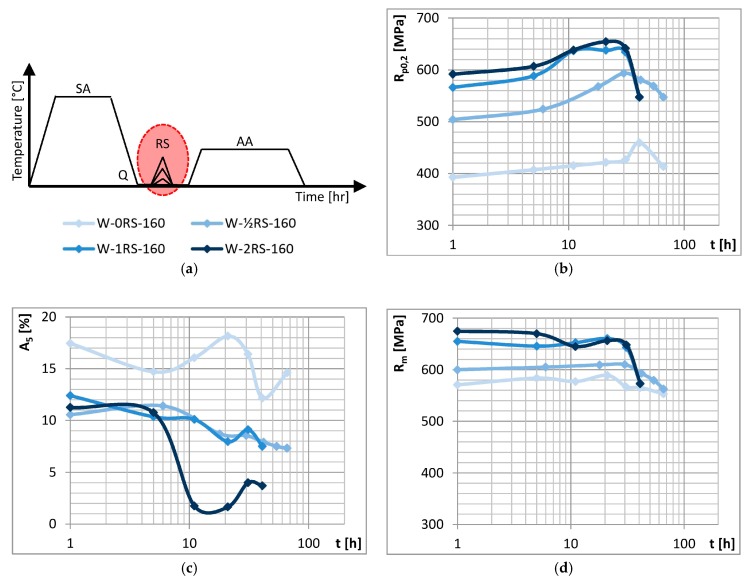
(**a**) Schematic representation of the strain magnitude experiment and its results: (**b**) yield strength (**c**) elongation and (**d**) ultimate strength vs. time of AA.

Effects of the amount of strain were studied in specimens after experimental treatment (as shown schematically at top left in Figure 3) which involved different RS parameters. Besides the specimens which underwent no deformation (0RS), three series of specimens (½RS, 1RS, 2RS) were prepared with different numbers and/or amounts of reduction.

Figure 3 shows the ageing curves. All the curves comprise three portions which indicate different stages of ageing: under-ageing, peak-ageing and over-ageing. With increasing amount of strain, yield strength and ultimate strength become higher, whereas elongation decreases. However, this only holds up to a certain strain magnitude. Differences between the curves 0RS–½RS–1RS suggest that yield strength and ultimate strength rise gradually but elongation decreases from one to the next. For elongation, this only applies between 0RS and ½RS. No appreciable difference between elongation levels was found between ½RS and 1RS. When strain is increased even further, i.e., in 1RS and 2RS, no additional increase in yield strength and ultimate strength occurs. Elongation, however, does decrease. The fluctuation in elongation may be attributed to the recovery processes which accompany precipitation during AA.

**Figure 4:**
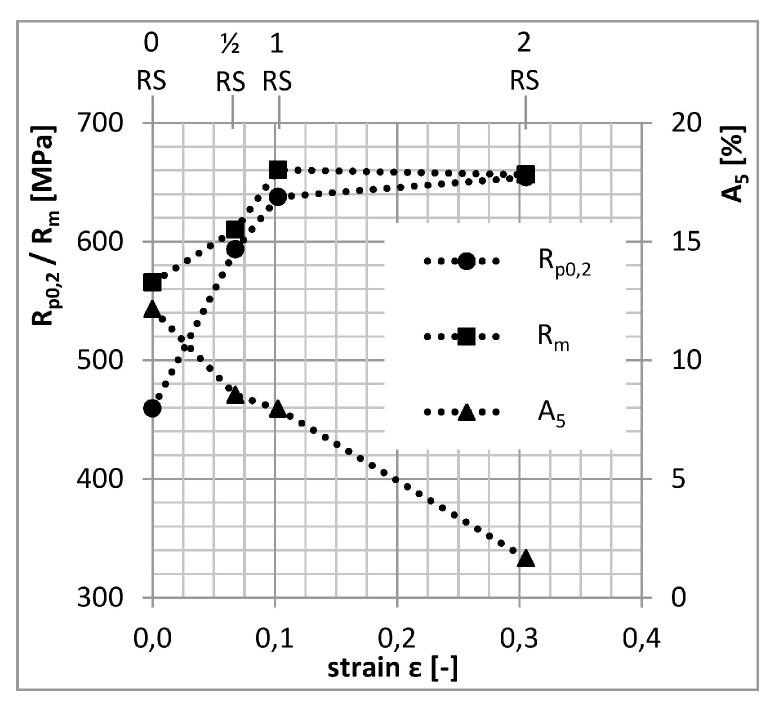
Peak-ageing mechanical properties vs. strain.

The effects of forming are best illustrated in the graph of mechanical properties after peak-ageing (for 0RS after 160 °C/41 h, for ½RS after 160 °C/30 h, and 1RS and 2RS after 160 °C/21 h) vs. the amount of strain (Figure 4). As the amount of strain exceeds ε = 0.103 (1RS), yield strength does not increase any further. What is more, ultimate strength slightly decreases, along with a drop in elongation. The cause of this is the loss of capacity for plastic deformation.

### 3.3. Temperature and Time of Artificial Ageing

**Figure 5:**
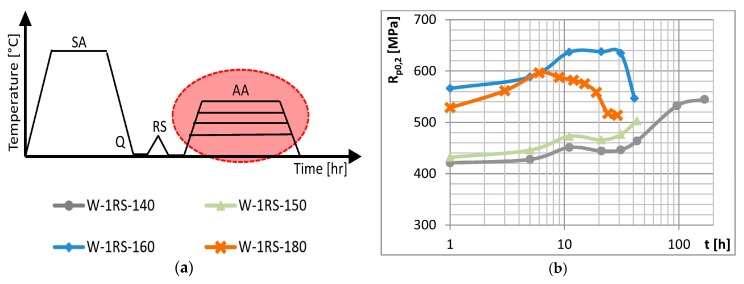

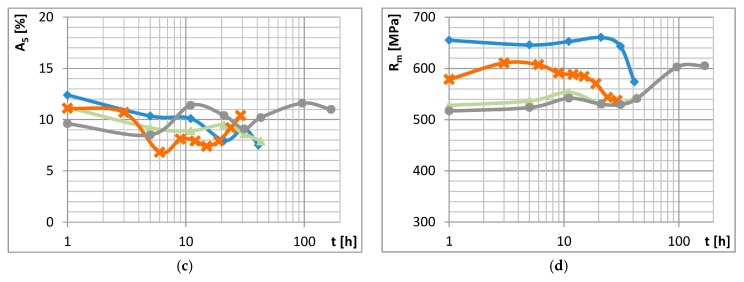
(**a**) Schematic representation of the AA conditions experiment and its results: (**b**) yield strength (**c**) elongation and (**d**) ultimate strength vs. time of AA.

Effects of the temperature and time of AA were studied in specimens after experimental treatment (as shown schematically at top left in Figure 5) which involved different AA parameters. The temperature of AA was 140, 150, 160 and 180 °C. The times of AA were 1 to 29 h for the highest temperature and 1 to 168 h for the lowest temperature.

**Figure 6:**
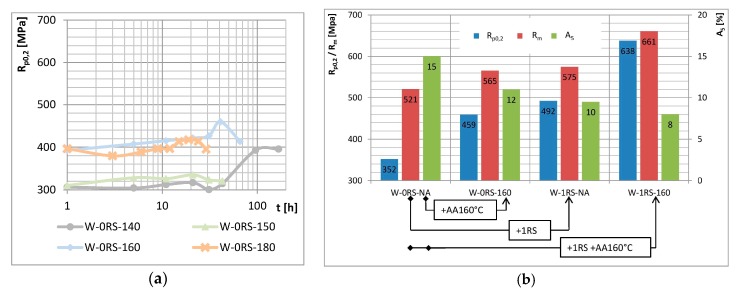
(**a**) Yield strength vs. time of AA for the condition with no deformation (0RS); (**b**) Mechanical properties of material of four selected conditions.

Figure 5 shows ageing curves in three graphs. Higher temperatures of AA lead to higher yield strength and ultimate strength. This only applies below a certain temperature. When specimens aged at 140, 150 and 160 °C are compared, increases in yield strength and ultimate strength are apparent. By contrast, both strength levels decreased in specimens aged at 160 and 180 °C. In those aged at 140 and 150 °C peak-ageing was not achieved within the times used. In specimens aged at 160 °C, the highest yield strength is obtained after 21 h. In those aged at 180 °C, it is after 6 h. This indicates that peak-ageing shifts towards shorter times as the temperature increases. The temperature of AA, unlike the strain, has no significant impact on elongation. The elongation curves fluctuate with ageing times but they also overlap. The comparison between curves with deformation (0RS) and with deformation (RS) (Figure 6a) shows that their shapes and the trends they reveal remain the same. Peak-ageing is shifted towards shorter ageing times and deformation adds another 100 to 150 MPa of strength.

Final mechanical properties are quite remarkable. The material with the best final parameters was obtained in the following manner: solution annealing 500 °C/1 h + water cooling + rotary swaging + artificial ageing 160 °C/21 h; and exhibits mechanical properties as listed in Table 3. These values are unique, given the RS process. Unlike the SPD processes, such as ECAP and HPT, that can achieve similar or better mechanical properties [11,12,13,14,15], the RS process has the higher potential for industry use.

The properties attained result from synergies among strengthening mechanisms initiated by the experimental processing. There are contributions from mechanical working, i.e., grain size strengthening and strain hardening and from AA, i.e., precipitation strengthening. The interaction between them is readily visible in graph in Figure 6b. The values on the left characterize a specimen which has not undergone RS or AA. It was only processed using NA (Water-0RS-NA). Next to them, there are columns for a specimen without RS and with AA (Water-0RS-160). Further to the right, the columns relate to a specimen after RS and NA (Water-1RS-NA). The rightmost section shows the best values achieved (W-1RS-160, see Table 3). AA alone (arrow +AA160 °C) or rotary swaging alone (arrow +1RS) leads to an increase in yield strength by 107 MPa and 140 MPa respectively, ultimate strength by 44 MPa and 54 MPa, respectively, and to a reduction in elongation by 3% and 5%, respectively. Clearly, the mechanical properties of material in the best condition are not just sums of contributions from individual factors. The results are larger than that (638 MPa > 352 + 107 + 140; 661 MPa > 521 + 44 + 54; 8% > 15 − 3 − 5).

### 3.4. Microstructure Characterization

#### 3.4.1. SEM Observation

**Figure 7:**
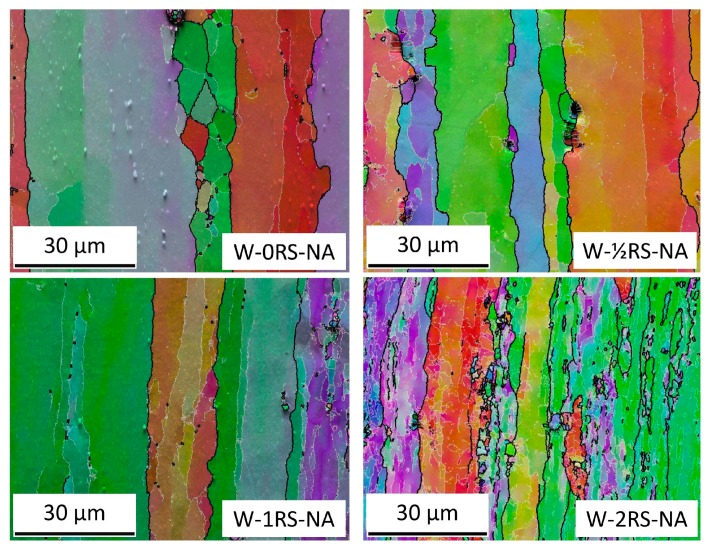
EBSD images where HAGB are shown in black and LAGB in white.

**Figure 8:**
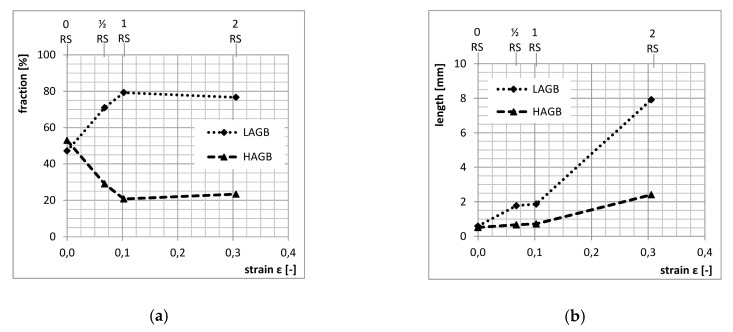
Quantitative evaluation of LAGB and HAGB: (**a**) fraction of LAGB and HAGB vs. strain and (**b**) total length of LAGB and HAGB vs. strain.

The specimens selected for SEMEBSD analysis had longitudinal sections with different amounts of strain (0RS/½RS/1RS/2RS). EBSD images (Figure 7) clearly show the microstructural evolution with increasing strain. The fractions of LAGB and HAGB and their total lengths in the field under analysis were measured. It is apparent that the initial fraction of LAGB at 50:50 increases with strain to approx. 80% in the 1RS condition (Figure 8a). Additional strain (2RS condition) does not alter this value substantially. The total length of grain boundaries, for both LAGB and HAGB, is constantly growing between these sequences (Figure 8b).

In graphs in Figure 4 and Figure 8a, the trends characterizing yield strength, ultimate strength and fraction of LAGB vs. strain are identical. With increasing amount of strain, the fraction of LAGB increases all the way to 80%. At the same time, yield strength and ultimate strength increase as well. The highest level is obtained with the amount of strain identified as 1RS, which led to the specimen with the best properties. With additional strain (2RS), the fraction of LAGB or yield strength and ultimate strength do not change appreciably. However, elongation drops, due to much higher dislocation density.

#### 3.4.2. TEM Observation

The specimens which were selected for TEM observation were from transverse cross sections for various ageing conditions (W-1RS-140/21—under-ageing; W-1RS-160/21—peak-ageing; W-1RS-180/19—over-ageing) and were intended to provide insight into phase development during ageing. An additional specimen, W-0RS-160/21, was chosen to provide a comparison between conditions with and without deformation.

**Figure 9:**
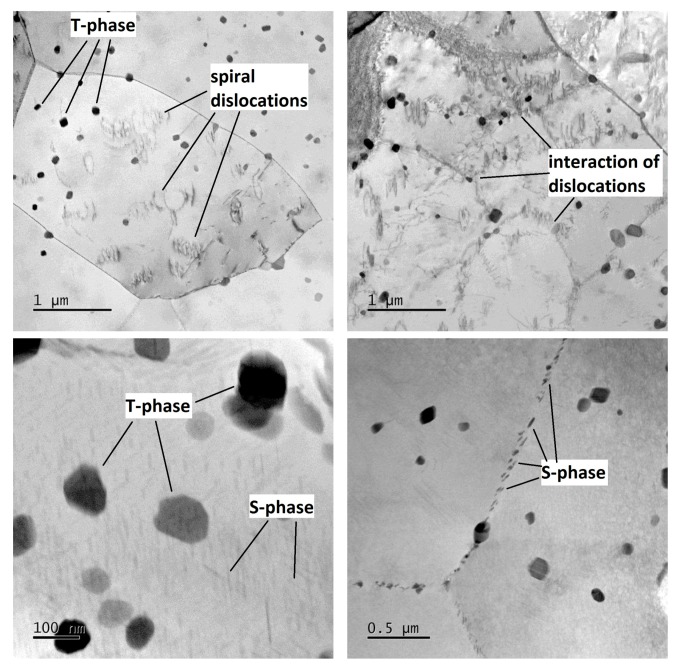
Micrographs of specimen W-0RS-160 at different magnification with marked dislocations, T-phases, and S-phases.

**Figure 10:**
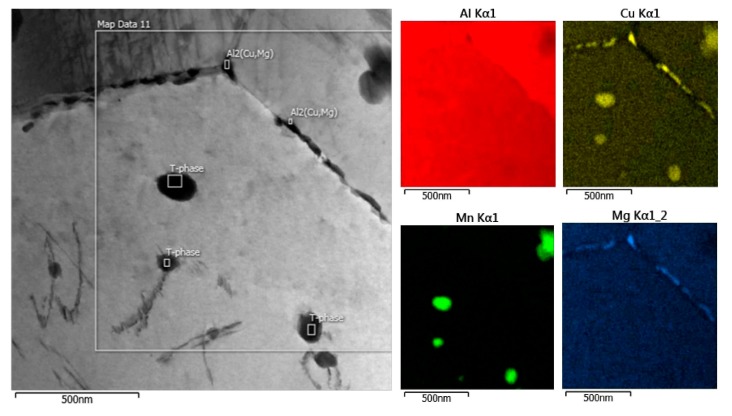
EDS maps of specimen W-0RS-160.

**Figure 11:**
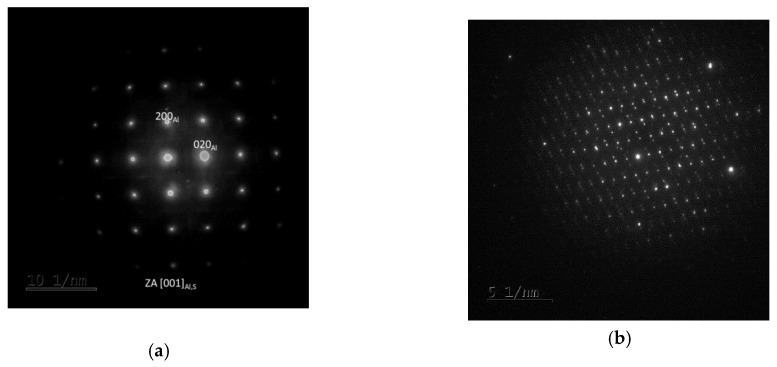
(**a**) Selective electron diffraction pattern: Al matrix in the zone axis [001], the Al matrix is shown as clear spots and a square pattern, whereas the S-phase is represented by dim crosses among the clear spots; (**b**) Diffraction pattern of the T-phase.

The microstructure of the undeformed specimen W-0RS-160 contains individual dislocations which interact with each other and with precipitates (Figure 9). There are three types of precipitates in the material. Measurement of chemical composition (Figure 10) and evaluation of diffraction patterns (Figure 11) revealed that the dark particles are the T-phase (Al_20_Cu_2_Mn_3_). They are typically found within grains, having an oval shape and a size of 10–100 nm. Rare occurrences include rods with lengths up to 1 μm. T-phase in the form of rods is documented in [21].

As the T-phase does not contain Mg, its surroundings offers favourable conditions for the S-phase (Al_2_CuMg) to form, which is rich in Mg. The S-phase forms either around the T-phase or along grain boundaries. Along grain boundaries, this phase is present in the form of particles smaller than 1 µm. Under certain conditions, S-phase takes the form of needles which are coherent with the Al matrix and oriented in <100> direction. In this specimen, precipitation within grains was not extensive. This is evidenced by the diffraction patter in Figure 11. Here, the S-phase produces indistinct indications rather than clear diffraction spots.

**Figure 12:**
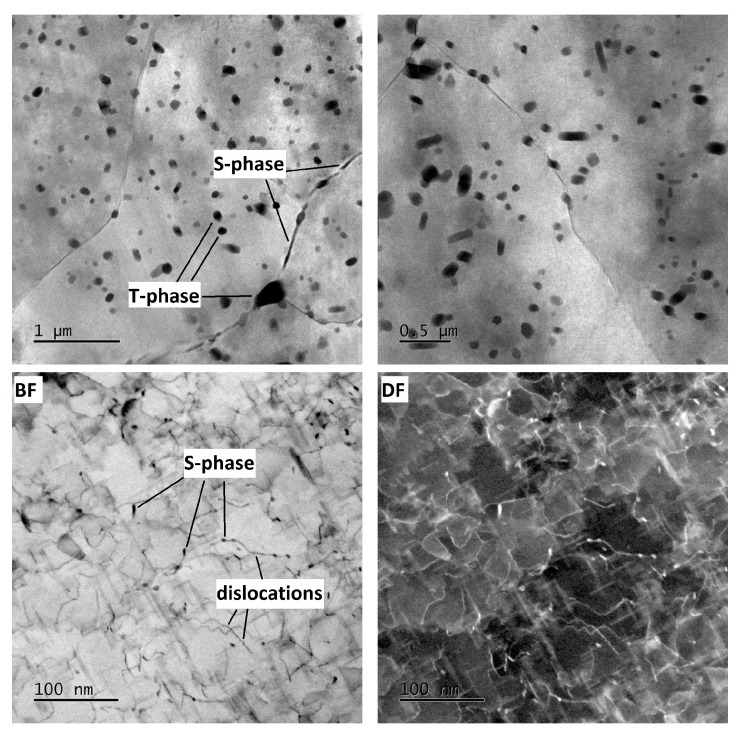
Micrographs of specimen W-1RS-160 at different magnification with marked dislocations, T-phases and S-phases.

In the mechanically-worked specimen W-1RS-160 in peak-ageing condition, under diffraction conditions suitable for observing dislocations, the image of microstructure is not clear enough for assessment. Figure 12 taken in the bright field (BF) and the dark field (DF) shows dislocations with a very high density in the matrix. As with W-0RS-160, almost no isolated dislocations were found here. Using chemical and diffraction analysis, the presence of the same particles was confirmed as in W-0RS-160, namely T-phase and S-phase in two shapes. The precipitation of S-phase needles is more extensive in W-0RS-160, possibly due to having been driven by rotary swaging. Morphologies of the needles are distinctly visible in dark field images. In Figure 12, the foil is oriented perpendicularly to the [100] direction, and therefore needles of the S-phase can be seen in two perpendicular directions. The needles in the third direction, perpendicular to the plane of the image, appear as dark spots. These images also show interaction between dislocations and these needles. S-phase particles on grain boundaries are larger than in the previous case.

**Figure 13:**
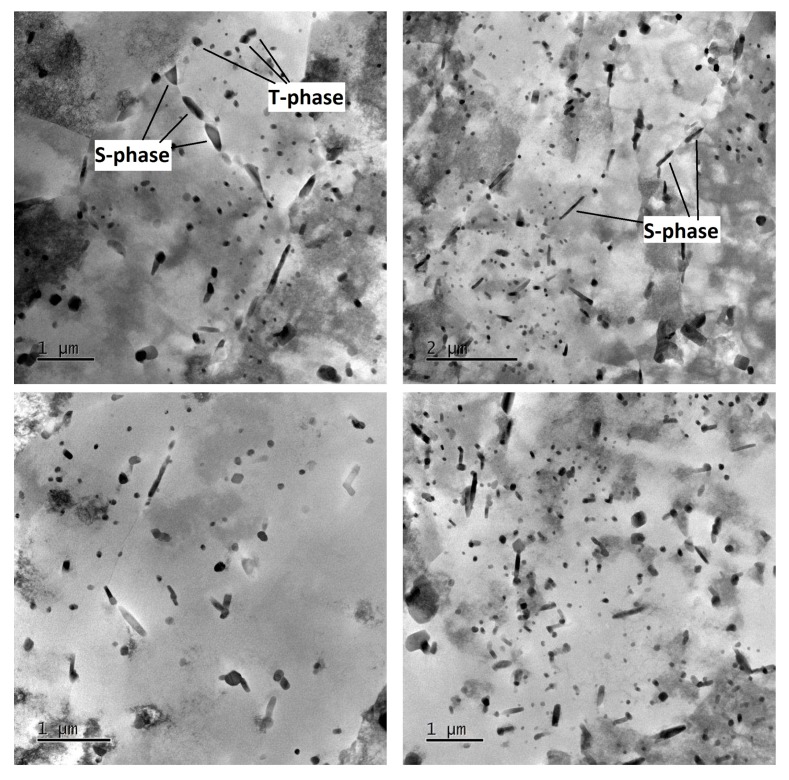
Micrographs of specimen W-1RS-140 at different magnification with marked T-phases and S-phases.

In the under-aged W-1RS-140 specimen, coherent needle-like precipitates of the S-phase were not present. Apart from T-phase precipitates, relatively coarse S-phase precipitates within grains were found (Figure 13). Here, the S-phase precipitates on grain boundaries were the largest among all the material conditions.

**Figure 14:**
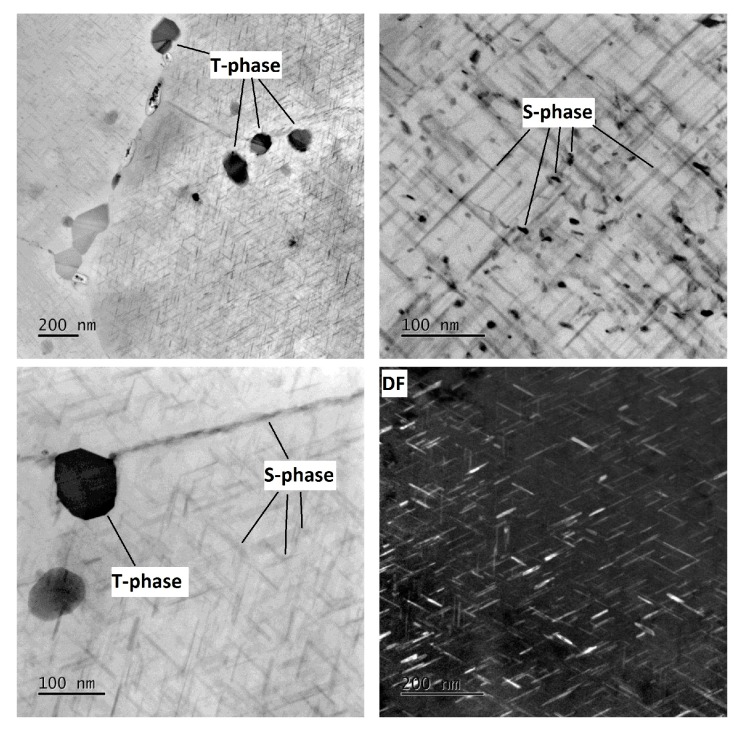
Micrographs of specimen W-1RS-180 at different magnification with marked T-phases and S-phases.

**Figure 15:**
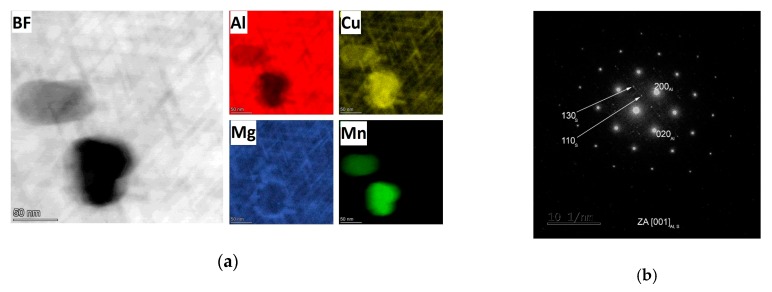
(**a**) EDS maps of specimen W-1RS-180; (**b**) Selective electron diffraction pattern of the Al matrix in the zone axis [001]: the Al matrix is represented by bright spots in a rectangular pattern, The S-phase is shown as dimmer spots between the bright ones.

In the over-aged W-1RS-180 specimen, T-phase precipitates and S-phase precipitates on grain boundaries are present. Intensive precipitation of needle-like S-phase particles in the grain interior occurred, Figure 14. General tilt reveals three orientations of S-phase needles. When the electron beam is parallel to [001] direction of the matrix the elongated shape only shows two orientations of needles in the plane perpendicular to the incident electron beam. In needles oriented in the third direction, the longitudinal axis is parallel to the electron beam, i.e., perpendicular to the image plane. Therefore they are projected as small oval particles. High-magnification chemical composition maps show that S-phase needles contain increased amounts of Cu and Mg (Figure 15a). The selective diffraction pattern (Figure 15b), which is identical to that reported in [22], shows clear spots of the matrix in [001] orientation and dimmer spots from coherent precipitates in the matrix.

It is obvious that samples in different conditions from under-ageing to over-ageing have different microstructure in terms of the shape, size, and distribution of phases. In under-ageing condition, there are present just coarse T-phases and coarse S-phase, both in an oval shape with size up to 100 nm. The T-phases have occurred within grains, the S-phase within grains and on grain boundaries too. The ageing has no significant influence on the morphology of T-phases. In peak-ageing and over-ageing condition, there are present the T-phases in a similar shape, size, and distribution. The ageing has a significant influence on precipitation of S-phase. Needle-like S-phases within grains are present in the peak-ageing condition in a small amount. These needles are present in the over-ageing condition in a large amount. These needles of S-phase have probably the most substantial effect on improving the mechanical properties.

### 3.5. DSC Analysis

**Figure 16:**
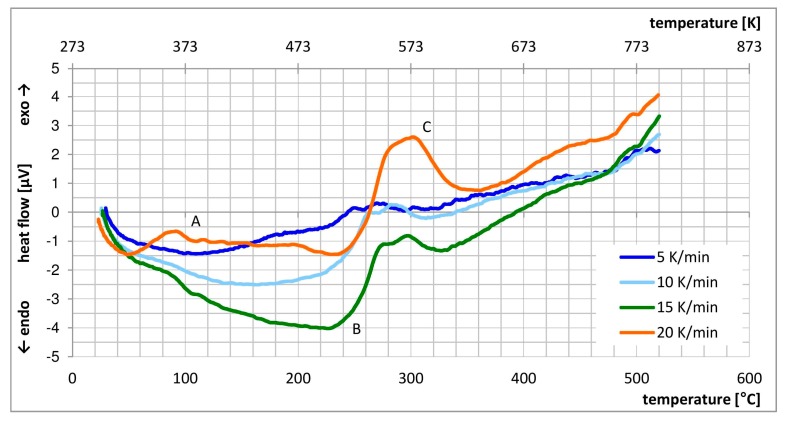
DSC curve used for finding the solution annealing temperature.

Figure 16 presents DSC curves for EN AW-2024 alloy for four different heating rates: 5, 10, 15 and 20 K/min. DSC curves include several exothermic and endothermic peaks, indicating precipitation and dissolution processes. At the peak temperature, two competing processes take place. The driving force for precipitation decreases due to reduces super-saturation of SSSS and the diffusivity of individual elements increases. In precipitation-hardenable aluminium alloys, the general rule says that precipitation is an exothermic process, whereas dissolution is an endothermic one [23].

In agreement with studies [24,25], three peaks can be distinguished: A—formation of Cu-Mg clusters and GPB2 zones; B—dissolution of GPB2 zones; C—precipitation of the S-phase. With increasing heating rate, the peaks become more prominent and shift towards higher temperatures (Table 4). The reason for this is that at higher heating rates, the reaction (precipitation/dissolution) must finish within a shorter time, and therefore becomes more intensive. It follows that these reactions are thermally-activated and their progress is dictated by the heating rate. Type A peak is only distinct when the highest heating rate is used. Type B peak can be found in the two highest heating rates. Type C peak is visible upon all four heating rates.

## 4. Conclusions

In this study, the effects of rotary swaging and artificial ageing parameters on the properties of EN AW-2024 alloy were explored. Microstructure, its evolution, and associated processes were described.

The best mechanical properties were obtained with solution annealing at 500 °C for 1 h followed by water cooling and rotary swaging (true strain ε = 0.103) and final artificial ageing in a furnace with forced circulation of atmosphere at 160 °C for 21 h.

The sequence led to exceptional mechanical properties: yield strength R_p0.2_ = 638 MPa, ultimate strength R_m_ = 661 MPa, elongation A_5_ = 8%, reduction of area Z = 17.6%. These values were achieved thanks to synergistic effects of rotary swaging and artificial ageing. This sequence of operations is well-suited for practice.

Using SEM and EBSD, it was found that the forming process increased the total length of low-angle and high-angle boundaries, and changed their ratio from the initial 50:50 to approximately 80:20.

TEM observation revealed that the microstructure contained T-phase (Al_20_Cu_2_Mn_3_) and S-phase (Al_2_CuMg) precipitates. T-phase is found mostly within grains, being oval-shaped with a size of 10–100 nm. S-phase precipitates are on grain boundaries as well as in the grain interior, where it has two morphologies. One such morphology is small oval particles and the other is coherent needles oriented along the <100> direction.

## Figures and Tables

**Table 1:** Chemical composition of EN AW-2024 alloy (wt. %).

Component	Cu	Mg	Mn	Zn	Ni	Ti	Cr	V	Si	Fe	Al
Amount [%]	4.35	1.46	0.66	0.14	0.084	0.055	0.02	0.015	0.33	0.23	rest

**Table 2:** Rotary swaging parameters.

Designation	Initial Diameter	Diameter after 1st Pass	Diameter after 2nd Pass	True Strain *
0RS	12 mm			0
½RS	12 mm	11.6 mm		0.068
1RS	12 mm	11.4 mm		0.103
2RS	12 mm	11.4 mm	10.3 mm	0.306

* True strain ε is a dimensionless quantity and is defined as $\varepsilon =\ln\frac{S_{0}}{S}$, where *S*_0_ is the initial cross-section area and *S* is the final one.

**Table 3:** Mechanical properties of W-1RS-160/21 specimen.

R_p0,2_	R_m_	A_5_	A_g_	Z
(MPa)	(MPa)	(%)	(%)	(%)
638	661	8	3	17.6

**Table 4:** Maximum temperatures for individual peaks (in °C).

Peak	5 K/min	10 K/min	15 K/min	20 K/min
A				92
B			227	230
C	270	282	297	302
